# Protocells Either Synchronize or Starve

**DOI:** 10.3390/e27020154

**Published:** 2025-02-02

**Authors:** Marco Villani, Roberto Serra

**Affiliations:** 1 Department of Physics, Informatics and Mathematics, Modena and Reggio Emilia University, 41121 Modena, Italy; rserra@unimore.it; 2 European Centre for Living Technology, 30123 Venice, Italy; 3 Institute of Advanced Studies, University of Amsterdam, 1012 WX Amsterdam, The Netherlands

**Keywords:** self-reproduction, self-replication, diffusion rate, Fick’s law, transmembrane diffusion, chemical kinetics

## Abstract

Two different processes take place in self-reproducing protocells, i.e., (i) cell reproduction by fission and (ii) duplication of the genetic material. One major problem is indeed that of assuring that the two processes take place at the same pace, i.e., that they synchronize, which is a necessary condition for sustainable growth. In previous theoretical works, using dynamical models, we had shown that such synchronization can spontaneously emerge, generation after generation, under a broad set of hypotheses about the architecture of the protocell, the nature of the self-replicating molecules, and the types of kinetic equations. However, an important class of cases (quadratic or higher-order self-replication) did not synchronize in the models we had used, but could actually lead to divergence of the concentration of replicators. We show here that this behavior is due to a simplification of the previous models, i.e., the “buffering” hypothesis, which assumes instantaneous equilibrium of the internal and external concentrations of those compounds which can cross the cell membrane. That divergence disappears if we make use of more realistic dynamical models, with finite transmembrane diffusion rates of the precursors of replicators.

## 1. Introduction

It is well known that the process of cell division (fission) is of the utmost importance for every living species, either unicellular or multicellular. In this paper we will consider only the case where a single mother cell gives birth to two daughters; in order to assure that every daughter cell receives a full copy of the mother’s genetic material, cell fission is usually preceded by duplication of its DNA which, in present-day cells, is guaranteed by sophisticated control mechanisms [1]. However, it is highly unlikely that such control mechanisms were in place in the early days of primordial protocells.

Protocells are entities that resemble in some way, but are much simpler than, present-day cells and that are supposed to have been their predecessors. Several interesting intermediate results have been obtained in the laboratory [2,3,4], however full-fledged protocells, able to continuously generate several successive generations, have not yet been achieved.

While very many different hypotheses about the “architecture” of protocells have been suggested, most research works are based on lipid vesicles, which are spontaneously formed under a broad set of conditions in aqueous solutions of amphiphiles [5,6,7,8,9]. Such lipid vesicles resemble cells in that their aqueous interior is surrounded by an approximately spherical membrane composed of a lipid bilayer. Moreover, if further lipids are supplied, the size of vesicles can grow and, under some experimental conditions, their splitting has been observed [2,10,11,12,13]—a process which is reminiscent of cell fission.

Actual “wet” experiments are expensive and require long times; therefore, mathematical and computational models are extremely important to indicate directions of research, to test the suitability of different hypotheses, and to point out major problems which need to be addressed [14,15,16]. In this paper we will indeed consider broad classes of mathematical and computational models of protocells, all based upon semipermeable lipid vesicles which will be assumed to spontaneously undergo fission when they reach a certain size. Moreover, it will also be assumed that each protocell hosts in its internal water phase some chemicals (“replicators”) which are able to collectively self-replicate, and that some replicators also increase the rate of growth of the membrane (e.g., by catalyzing the synthesis of its amphiphiles). For simplicity, we consider a single type of lipid, so the set of replicators determines the identity (i.e., the properties) of the protocell itself, and can be regarded as its proto-genetic material. In general, we will neglect other chemicals which might be found inside the protocell, which are not directly involved in the self-replication nor in the synthesis of membrane lipids.

Two different processes take place in these vesicles, i.e., (i) cell reproduction by fission and (ii) replication of replicators. One major problem is indeed that of assuring that the two processes take place at the same pace, i.e., that they synchronize (since fission gives rise to two offsprings, synchronization is achieved if and only if also replication leads to doubling the quantity of replicators). If cell reproduction were much faster than duplication, the proto-genetic material would be increasingly diluted through generations, while in the opposite case its quantity would continue to increase and accumulate in cells. In both cases no sustainable growth of a population of protocells would take place, and therefore life could not emerge in a robust way; even if some protocell were able to reproduce, this property would be lost by its descendants.

It cannot be taken for granted that the rates of these two different kinetic processes are born identical, but it is interesting to understand under which conditions, in an evolving population, they can converge to a common value, generation after generation. In this case, their synchronization might have spontaneously taken place in early (proto)life.

In a series of previous works, we used simplified mathematical and computational models to address this issue. The goals, results, and limitations of these models have been discussed in several papers and in a book [17]; in Section 2 of this paper, we will briefly summarize their main features, referring the interested reader to those works for further information. In order to avoid filling the reference list with many self-citations, we limit ourselves here to mentioning the two foundational papers [18,19], where the physical hypotheses are discussed in depth, and the book [17], where one can find proper references to most previously published papers. We will also directly cite, where necessary, our more recent works.

In these models, protocells are supposed to be semipermeable, and indeed a Boolean approximation is introduced, thus sharply distinguishing those chemicals which can cross the membrane from those which cannot. Diffusion is assumed to be extremely fast in the bulk, both inside the protocell and in the external environment, so there is no need to consider concentration gradients which instantaneously vanish, and space is homogeneous, both inside and outside. Moreover, also transmembrane diffusion of the species which can cross the membrane (in the following called also “permeable species”) is assumed to be instantaneous, so that their internal and external concentrations are the same (the “buffering hypothesis”, in chemical jargon). Note that reactions which take place inside the protocell can make the internal and external compositions different, if they involve non permeable species. Note also that the external environment acts as a large reservoir, and that the concentrations of various chemicals are not affected by the activities of the protocell.

The models host two kinds of kinetic equations, those which rule the growth of the membrane and those which describe the interactions among replicators. It may also happen that the growth of the population stops, a condition which we refer to as the “starvation” of the population, although no explicit model of cell death is introduced: we simply say that a population starves when it stops (or never starts) growing. It may happen that the same equation system supports either growth or starvation, depending upon the values of some parameters. We refer to these kinds of equations as “synchronizing”, since synchronization can actually be achieved, for some parameter values.

We have examined different models, analyzing different types of linear and nonlinear kinetic equation systems, and it was shown that synchronization actually emerges under a surprisingly wide set of different hypotheses, without resorting to any specific evolutionary mechanism.

In these models, fission takes place when the size of the cell reaches a threshold value, giving rise to two identical daughters, whose size equals the initial size of their mothers. When synchronization is achieved (this is literally true only in the limit t → ∞), the initial concentration of replicators in the daughters is also identical to that of their mothers, therefore they take the same time to reach the threshold size for fission; all the generations tend to become identical, and to have the same duration. Therefore, the doubling times tend to be equal, so that the growth of the population is exponential, and this implies, as first observed by [20], that selection (which takes place only when population growth is somehow limited) is of the Darwinian type (survival of the fittest), even when the kinetic equations for replicators are nonlinear.

An interesting phenomenon has also been reported in some cases, when the time interval needed to reach the fission threshold changes from one generation to the following one, doing so in a cyclic way, so that the duplication time of generation *k* is the same as that of generation *t + k* (in the long-time limit). This is still a kind of (periodically oscillating) synchronization, and it has therefore been called super-synchronization.

In a few interesting cases, synchronization can be analytically proven, in other cases it can be verified by numerical simulations. It can thus be shown that it is robust with respect to random fluctuations of the size of the newborn cells and of the value of the splitting threshold.

The results discussed in this paper refer to models where the replicators are all in the internal water phase, but synchronization has also been proven for different protocell architectures, including the so-called “surface reaction models” (SRMs) (for clear reasons, the models which are studied in this paper have been called Internal Reaction Models—IRMs), where the replicators are located in the lipid membrane, and simplified models of the GARD kind (where the replicators are themselves lipids) [21,22] and of self-replicating micelles [18].

This is good news; synchronization can be an emergent phenomenon under a broad set of hypotheses about the model equations and architectures, and it may have allowed sustainable protocell growth before the onset of the sophisticated checkpoints of evolved cells. However, there is an important exception, which is observed when the growth rate of replicators is (in a sense which will be precisely defined in Section 2) “too fast”, leading to divergence of the concentration of replicators.

For example, in the simplified case of a single replicator, this happens when the growth rate of the mass of the lipid container *C*, *dC/dt*, is proportional to the quantity of that replicator *X*, while *dX/dt* depends upon *X*^2^ (see Section 2 for more precise details). This may seem quite an odd hypothesis when there is a single type of replicator, but quadratic terms are quite often found in models with mutual catalysis of different replicators—and the same type of divergence is also observed in those models. Therefore, the lack of synchronization in case of quadratic or higher order kinetic terms in the equations is not only a point of mathematical rigor, without relevance for the hypothetical events of early life, but it actually limits the range of synchronizing models.

Therefore, we analyzed with care what happens when the order of the kinetic equations approaches two from below, observing that the internal quantities of replicators increase sharply, while duplication times approach zero: the growth of the internal concentration diverges, the time to duplication vanishes.

As mentioned above, one of the hypotheses of the class of models which had been analyzed is the “buffering” of the concentrations of the permeable species, which corresponds to assuming infinitely fast transmembrane diffusion. This hypothesis is quite frequent in chemical kinetics, when diffusion is much faster than the other dynamical processes which take place. But when one such process becomes infinitely fast, as in the case of the increase in the concentration of the replicators, then the buffering hypothesis loses its bases.

One should then look at the behavior of slightly different models, where a finite diffusion rate is introduced. We do so, as described in Section 3 below, by explicitly taking into account precursors of the replicators, which can cross the membrane with finite diffusion rates while the membrane is impermeable to replicators. It is extremely interesting and satisfying to remark that this simple modification suffices to get rid of all the observed divergences, so that every model we have considered actually synchronizes or supersynchronizes (unless starvation occurs).

The outline of the paper is as follows. In Section 2, we quickly summarize the “buffered” models we use and previous results about synchronization, showing also that it is not achieved when the kinetic equation for the replicators are quadratic. In Section 3, we introduce models with finite diffusion rates, showing in a simple one-dimensional case that divergence is removed, and then we analyze a number of different models, showing that in all the cases which have been examined there is no divergence. Finally, Section 4 provides some further comments.

## 2. The Buffered Models

In this section we briefly summarize previous works. Interested readers can find further information and in-depth discussions of the model features in [17] and in the original papers quoted there. The protocell is assumed to be spherical, with an aqueous interior surrounded by a membrane, composed of a single type of lipid, whose thickness is *δ*. If *r* is the radius of the internal part, its surface *S* and its volume *V* are obviously also determined. The volume *V_M_* of the membrane equals the difference between the volume of a sphere with radius *r + δ* and that of a sphere of radius *r* which, when *δ << r*, can be approximated by *Sδ*. The membrane is homogeneous, with volume density *ρ*, so the total quantity of lipids in the protocell membrane, *C = ρV_M_* ≅ *ρδS*, provides a measure of its size (for a large cell with a thin membrane, *V* is approximately proportional to *C*^3/2^, and *S* is approximately proportional to *C*). The hypothesis of a spherical protocell thus allows us to relate in a simple way the size of the protocell to the outcomes of the kinetic equations (remember that some replicators catalyze the synthesis of the membrane lipids). The splitting processes, where deformations from a spherical shape necessarily take place, are supposed to be fast with respect to the protocell growth, so they are regarded as instantaneous and they are not explicitly modeled. Splitting takes place (at a fixed value *C = θ*), giving birth to two identical offsprings. We usually suppose that no lipids are lost during splitting (other hypotheses may be - and have been - made; they do not modify the validity of the conclusions about synchronization); in order to comply with the spherical shapes of the newborn cells, one must admit that there is a loss of about 30% of the total internal volume.

The protocell is an open system, with a semipermeable membrane which prevents transmembrane diffusion of some chemicals. It is placed in a large external volume (a reservoir), whose concentrations are not affected by exchanges with the protocells. Those chemicals which can cross the membrane, i.e., the permeable chemicals, are buffered, so their internal volume concentrations always equal their (constant) external values.

In these models, the dynamics of a protocell between its birth and its fission are ruled by ordinary first-order differential equations, which describe how the quantities of replicators ***X*** = (*X*_1_…*X_N_*), and the quantity of lipid *C* change in time:

(1) $$\left\{\begin{array}{c} \frac{dC}{dt}=f\left(C,\vec{X}\right)\\ \frac{d\vec{X}}{dt}=g\left(C,\vec{X}\right) \end{array}\right.$$

It is usually assumed (i) that the kinetic equations for the replicators are given by the law of mass action, i.e., that they are proportional to the expected frequency of encounters between their types and (ii) that rate-limiting terms, which could play a role when products accumulate, can be neglected before fission occurs.

Note that Equation (1) involves quantities, while the more familiar equations of chemical kinetics are written in terms of concentrations, which are directly related to the frequencies of encounters. The same equations could of course also be written in terms of concentrations, without modifying our conclusions. We found that using quantities simplifies the approach in a case with changing volume like ours—therefore we usually resorted to quantities, although we sometimes also used concentrations [19].

The size of the mother cell when splitting takes place is fixed, as well as the initial size of the newborns. The volume concentrations of the replicators of the newborns are equal to those of the mother cell at division time. Equation (1) thus allows us to compute the relationship between the initial quantities of replicators at successive generations; if ***X***(*k*) denotes the initial quantities of replicators at generation *k*, they determine the discrete map which relates ***X***(*k + 1*) to ***X***(*k*). In order to prove synchronization, it is necessary to show that they tend to constant values as *k* → ∞; since the size of each protocell at the beginning of a new generation is fixed, this condition guarantees that the total quantity of replicators, in the pair of daughter protocells, is twice that of the mother.

The differences among various specific IRM models should be found in the different types of kinetic equations. Although we have also considered terms where the container growth rate depends upon some power of the replicators [17], in order to limit the number of different cases of study, in this paper we will always assume that *dC/dt* increases linearly with ***X*** (it can depend upon a single component *X_k_*, or upon a sum of such linear terms).

We analyzed discrete maps which are obtained from different types of linear and nonlinear kinetic equations for the replicators, and it was possible to show that synchronization actually emerges under a surprisingly wide set of different hypotheses, without resorting to any specific evolutionary mechanism, provided of course that the term *g*(*C*,***X***) provides a significant increase in the quantity of replicator in a generation. This somewhat vague statement can be given precise meanings when the form of the kinetic equations is specified. For example, in the case of a system of linear equations for the replicators such as

(2) $$\frac{d\vec{X}}{dt}=M\vec{X}$$

it is related to the sign of the real part of the eigenvalue with the largest real part of the *NxN* matrix ***M*** (when some entries *M_hk_* are negative, supersynchronization can sometimes be observed) [17], which must be positive to assure synchronization. In another interesting case, where the equations are based on the binary polymer model [23,24], it can be shown that synchronization depends upon the presence of so-called RAF sets [25,26].

In a few cases, synchronization can indeed be analytically demonstrated; in other cases, it can be verified by numerical simulations. As it has been anticipated in Section 1, synchronization is also robust with respect to random fluctuations of the size of the newborn cells and of the value of the splitting threshold, and it holds for different protocell architectures. But it does not always happen.

Let us consider the case where there is a single replicator *X*, whose rate of growth is an increasing function of the quantity *X*. If the growth law is linear, i.e., *dX/dt = ηX*, then there is synchronization if *η > 0*, otherwise starvation occurs. But the effective rates of autocatalysis can also depend upon nonlinear exponents. In general, with a single replicator X, the equation system [1] becomes (for the detailed form of Equation (3), in particular the dependence upon *V*^1−*n*^, see [17])

(3) $$\left\{\begin{array}{c} \dot{C}=\alpha X\\ \dot{X}=\eta V^{\left(1-\nu \right)}X^{\nu } \end{array}\right.$$

(recall that *V* is a known function of *C*).

In this case, it can be analytically proven [17] that there is no synchronization when *ν* = 2. While using a quadratic equation for a single type of replicator may look somewhat odd, it should be observed that the same lack of synchronization is observed also when different types of replicators quadratically interact (for example, in the case of only two types of replicators, there are terms proportional to *XY* both in the equation for *dX*/*dt* and *dY*/*dt*). These and other cases will be studied in Section 3; here we will show only the single-replicator case.

The *ν* = 2 value leads to divergence of the quantity of replicators in the protocell, and it cannot obviously be simulated. But we can simulate the behavior of Equation (3) for different *ν* values and look at how it changes when *ν* approaches 2 from below, i.e., in a region of parameter space where synchronization is observed.

**Figure 1:**
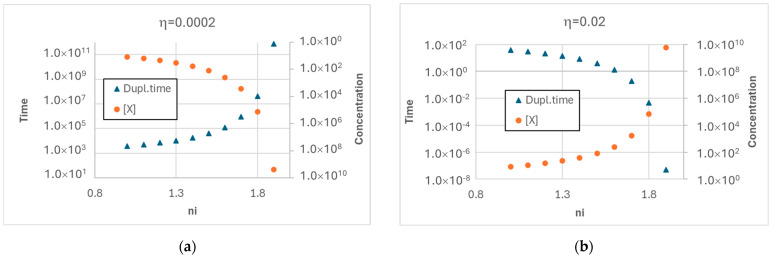
(**a**) Asymptotic time required for duplication (i.e., duration of a single generation) and asymptotic replicator concentration at the time of duplication, for low values of the *η* coefficient. In this situation, the growth of the exponent *ν* leads to increasingly lower values of replicator concentration and to increasingly higher duplication times (“starvation”). (**b**) The same variables for higher *η* value. In this situation, the growth of the exponent *ν* leads to increasingly higher replicator concentration values and ultimately to divergence, and consequently to increasingly shorter and ultimately vanishing duplication times. The *α* coefficient is 0.05 in both images.

In Figure 1, one can see two different behaviors. In Figure 1a, the asymptotic concentration of the replicator (which catalyzes the growth of membrane lipids) sharply decreases as the exponent ν approaches 2; consequently, the duplication times become longer and longer. As discussed above, although we do not model cell death, we call this behavior starvation, since the lack of replicators slows down cell duplication. The data in Figure 1b differ from the previous ones in that the growth rate of the replicator *η* is higher, while the coupling coefficient *α* with the protocell growth rate is unchanged. In this case, the asymptotic concentration of replicators increases faster and faster as the exponent *ν* approaches 2 (where it diverges, as it can be proven analytically [27]), while the time needed to reach the threshold value for fission (i.e., the duration of a generation) decreases to zero. In this case, a rapid increase in replicators does not seem to support a stable growth of the protocell population, a very counterintuitive outcome. It should also be said that in the literature, there are several models in which the reaction order is quadratic, or higher [15,22,28,29].

If the co-presence of more than one replicator is required to obtain another, we can write the scheme

(4) $$\left\{\begin{array}{c} X+Y+P_{x}\to 2X+Y\\ X+Y+P_{y}\to X+2Y \end{array}\right.$$

where *P_X_* and *P_Y_* are precursors of *X* and *Y*, respectively, which can cross the membrane. In the buffered models, their internal concentrations are the same as the external ones, which are constant. Therefore, the precursors can be omitted from the dynamic equations, and Equation (4) can be simplified to the following

(5) $$\left\{\begin{array}{c} X+Y\to 2X+Y\\ X+Y\to X+2Y \end{array}\right.$$

Therefore, if we suppose that the reaction rates are proportional to the frequency of encounters of the two reactants, and that the container growth is influenced only by *X*, the model equations for the 2D case of mutual catalysis are

(6) $$\left\{\begin{array}{c} \dot{C}=\alpha X\\ \dot{X}=\eta \prime V^{-1}XY\\ \dot{Y}=\eta \prime \prime V^{-1}XY \end{array}\right.$$

This model also shows divergence, like the one-dimensional model of Equation (3) when *ν* = 2, as it can be proven with analytical methods [17].

## 3. Finite Diffusion Rate

For reasons which have been extensively discussed in Section 1, the “buffering” approximation, which can provide useful results when transmembrane diffusion is much faster than chemical processes, can no longer claim validity when some reaction rates tend to infinite values. Therefore, we must leave it aside and consider the effects of finite diffusion rates. A way to do so is suggested by the notion of precursors which can cross the membrane (the “permeable” substances already mentioned in Section 1).

We will now suppose instead that precursors can cross the membrane, at a rate given by Fick’s law. Let us first consider the one-dimensional case, whose reactions are

(7) $$X+P_{x}\to 2X$$

Let *ϕ_x_* be the inflow rate of *Px* and let *D’* be its nonvanishing diffusion coefficient. Then, according to Fick’s law, *ϕ*_x_ is proportional to the product *D = D*’/*δ* (*δ* indicating the thickness of the membrane) times the membrane area S times the difference between the external concentration P* (which remains constant in the reservoir) and the concentration *[P_x_] = P_x_/V* in the internal water phase. The case corresponding to Equation (3) is therefore ruled, when diffusion rate is finite, by the following system:

(8) $$\left\{\begin{array}{c} \frac{dC}{dt}=\alpha X\\ \frac{dX}{dt}=\eta V{\left(\frac{X}{V}\right)}^{\nu }\frac{P_{x}}{V}=\eta {\left(V\right)}^{-\nu }{\left(X_{i}\right)}^{\nu }P_{x}\\ \frac{dP_{xi}}{dt}=DS\left(P^{*}-\frac{P_{x}}{V}\right)-\eta {\left(V\right)}^{-\nu }{\left(X_{i}\right)}^{\nu }P_{x} \end{array}\right.$$

The explicit introduction of the exponent ν, as in Equation (3), allows for greater generality, summarizing more complex reaction systems as, for example, chemical chain reactions [30].

**Figure 2:**
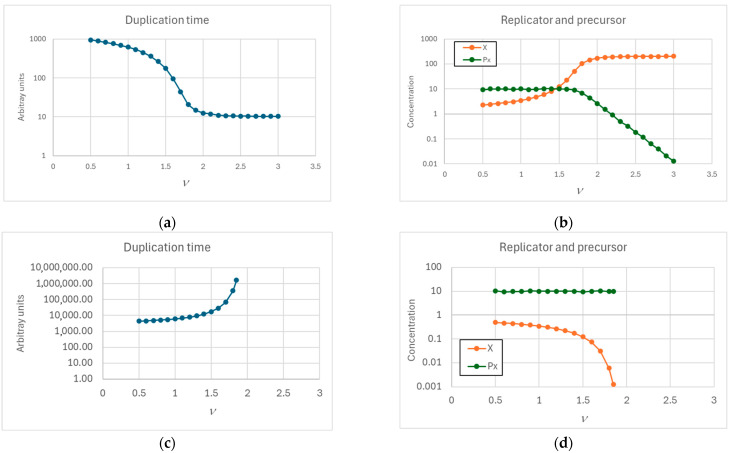
(**a**) Time needed for duplication (i.e., duration of a single generation) and (**b**) internal concentration of replicator and of the precursor at duplication time as exponent *ν* varies (*η* = 2.0 × 10^−4^, *α* = 10^−2^, *D* = 10^−14^). (**c**,**d**) The same, but with coefficients that lead to starvation (*η* = 5.0 × 10^−5^, *α* = 10^−2^, *D* = 10^−14^).

As shown in Figure 2b, in the case of a protocell with finite diffusion through the membrane, an increasing efficiency of the replicator production reaction (high *η_i_* and increasing value of the exponent *ν*) is accompanied by a corresponding decrease in the internal asymptotic concentration of the precursor, while the asymptotic concentration of the product remains almost constant (and consequently so does the duplication time). This happens because the internal concentration of the precursor becomes so low with respect to the external one that the flow of material (dependent on the difference between the two concentrations) cannot significantly increase. This limit implies that the precursor cannot be replaced effectively, and its concentration decreases; the decrease slows down the production of the replicator, which in turn cannot keep up with excessively rapid duplication rates. For high *ν*, at a steady state, the replicator depends directly on the incoming precursor flow, which cannot grow above the indicated limit, hence the constancy of the replicator concentration.

As already commented, low values of ν lead to increasingly lower asymptotic values of replicator concentration (Figure 2d), with corresponding increasingly higher duplication times (Figure 2c).

In the case of mutual catalysis with finite diffusion, the reaction scheme is given by Equation (4), and the full model is

(9) $$\left\{\begin{array}{c} \dot{C}=\alpha_{x}X+\alpha_{y}Y\\ \dot{X}=\eta_{x}V^{-1}YP_{x}\\ \dot{Y}=\eta_{y}V^{-1}XP_{y}\\ \dot{P_{x}}=D_{x}S\left(P_{x}^{*}-\frac{P_{x}}{V}\right)-\eta_{x}V^{-1}YP_{x}\\ \dot{P_{y}}=D_{y}S\left(P_{y}^{*}-\frac{P_{y}}{V}\right)-\eta_{y}V^{-1}XP_{y} \end{array}\right.$$

**Figure 3:**
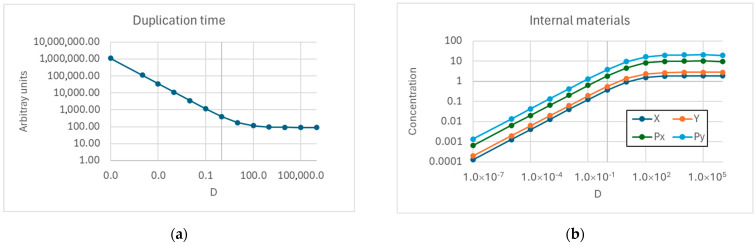
(**a**) Duplication time of a protocell composed of two replicators and two precursors that cross the membrane as the diffusion coefficient across the membrane varies—here, *D_x_ = D_y_*, *η_x_* = 9.0 × 10^−4^, *η_y_* = 10^−3^, *α_x_* = 5.0 × 10^−2^, *α_y_* = 5.0 × 10^−2^. (**b**) The concentrations at duplication time of replicators and precursors. There are no out-of-sync situations.

As shown in Figure 3, when starvation does not occur, synchronization is always observed, notwithstanding the significant variation in the diffusion coefficient across the membrane.

The same behavior also occurs in the case of systems composed of more than two replicators, where each replicator receives a positive catalytic contribution from at least one of the other replicators and where the precursor of each replicator is provided from the external environment.

We also considered the effects of finite transmembrane diffusion rates on models with a reaction order greater than 2; for example, in systems in which the catalytic intervention of two substances is required to carry out the reaction, in a way similar to the case of reactions needing enzymes and co-enzymes when substrate, enzymes and co-enzymes play similar roles [30]. We anticipate that in the case of a finite diffusion rate across the membrane, the divergences disappear.

The reaction system is then

(10) $$\left\{\begin{array}{c} \frac{dC}{dt}=\kappa V\left[X_{i}\right]=\kappa X_{i}\\ \frac{dX_{i}}{dt}=\eta V\frac{X_{j}}{V}\frac{X_{k}}{V}\frac{P_{xi}}{V}=\eta {\left(V\right)}^{-2}X_{j}X_{k}P_{xi}\\ \frac{dP_{xi}}{dt}=D_{Ai}SK\left({P_{xi}}^{*}-\frac{P_{xi}}{V}\right)-\eta {\left(V\right)}^{-2}X_{j}X_{k}P_{xi} \end{array}\right.$$

where it is assumed that each replicator is produced by a modification of its precursor, catalyzed by two other species.

The coupling between the catalyst pairs (*j*,*k*) and the catalyzed species *i* can be represented by a matrix, where a “1” appears in each row if species *j* (or *k*) participates in the catalysis of species *i*. The sum of the elements of each row is therefore equal to 2, while there are no constraints on the sum of the columns. In this work, we assume that only one pair of catalysts contributes to the formation of species *i*, and that there is only one trophic level (all necessary reagents—the precursors of the internal species—pass through the membrane and are therefore supplied by the external environment, where their concentrations are kept constant). The order of the reactions is equal to 3.

In the case of extreme uniformity (diffusion coefficients *D_Ai_* all exactly equal to each other, as well as the *η_i_* to each other), the distribution of the non-zero elements in the matrix is indifferent, and all the chemical species have the same exact behavior. This is obviously a very particular case, and in this work, therefore, we use an ensemble approach in which the networks belonging to each group share the same matrix, while the reaction constants change in the ensemble. We also performed other ensemble studies, keeping the values of the reaction rates constant, while randomly varying the coupling matrix; in some other cases, we varied both.

In the following, the variability of the kinetic parameter values involves multiplying the desired mean value times, or dividing by, a random coefficient with equal probability drawn from a uniform distribution in the interval [1.0, 3.0]. By observing the simulation results it is possible to draw some general regularities.

**Figure 4:**
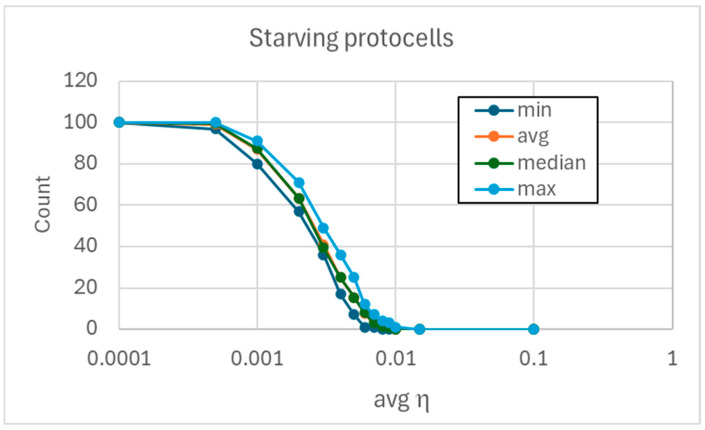
Minimum, mean, median, and maximum number of protocells that dilute as the mean value of the reaction coefficients varies (the constants *α_i_* are all set to 0.01). Statistics are calculated on 20 runs involving ensembles of 100 protocells, each protocell containing 15 replicators and the corresponding precursors; protocells that do not starve synchronize. The transition occurs in the narrow interval [0.0005, 0.01]; outside this zone all protocells have the same behavior (starvation, or synchronization).

In case of high *η_i_* coefficient values, the protocells always synchronize, regardless of the specific matrices used. In case of low *η_i_* coefficient values, the concentrations of the internal chemical species always decline—and consequently the precursors’ concentration values reach those of the external milieu—regardless of the matrices used (Figure 4).

**Figure 5:**
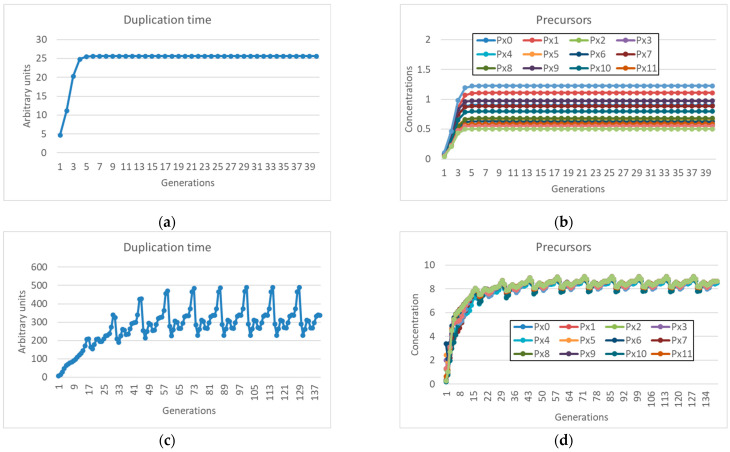
(**a**) The behavior of the duplication time in an example of synchronizing protocell, and (**b**) the corresponding concentrations of internal precursors (*α_i_* set to 0.01, random *η_i_* with mean close to 0.01). All measurements are taken immediately before the time of division. (**c**,**d**) The same behaviors in an observed case of supersynchronization (*α_i_* set to 0.01, random *η_i_* with mean close to 0.001—as commented in the main text, the variability of *η_i_* values involves multiplying the desired mean value by, or dividing by, a random coefficient drawn from a uniform distribution in the interval [1.0, 3.0]).

However, for intermediate values of the *η_i_* coefficients, it is possible that the protocells synchronize or not depending on the details of the matrix or on the particular values of the coefficients (Figure 4). In some situations, supersynchronization is possible, which is not observed in the case of high or low parameter magnitudes (Figure 5).

**Figure 6:**
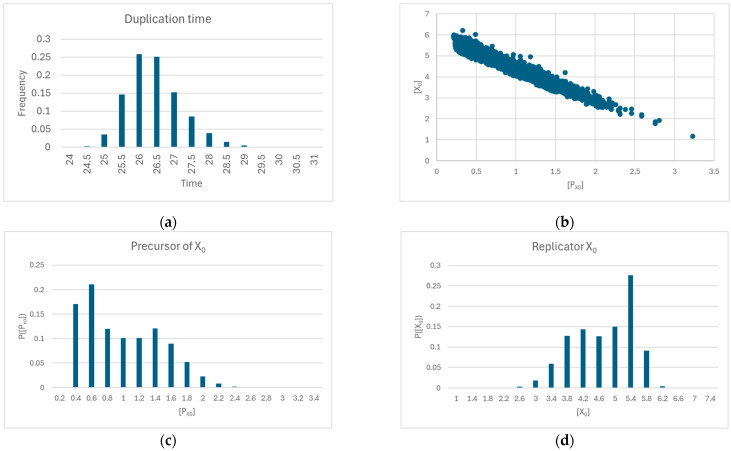
(**a**) Distribution of duplication times of an ensemble of 5000 protocells, composed of 15 replicators and their precursors (*α_i_* equal to 0.01, random *η_i_* with mean equal to 0.01). Only 15 protocells did not synchronize and therefore are not present in the distribution. (**b**) Relation at duplication time between the concentrations of a (randomly chosen) replicator and its precursor in the 5985 protocells that synchronized. (**c**) Concentration distribution of this precursor and (**d**) of the associated replicator.

In any case, by increasing the value of the reaction coefficients, protocells that do not synchronize begin to synchronize, confirming that the fundamental parameter is the magnitude of the reaction coefficients. Figure 6 shows some typical results (duplication times, and relationship between a replicator and its precursor) of protocells belonging to an ensemble in which reaction coefficients tending towards synchronization are used.

**Figure 7:**
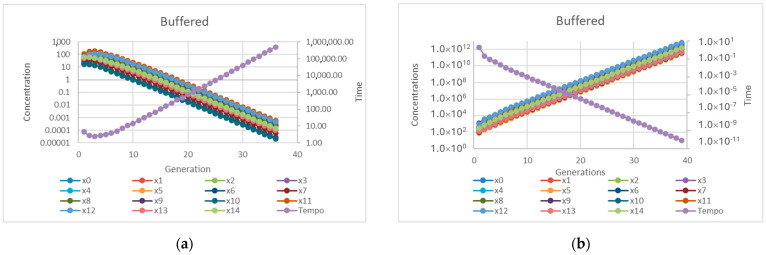
(**a**) Starvation of a protocell composed of 15 replicators: low *η_i_* (*α_i_* equal to 0.01, random *η_i_* with mean equal to 0.014). (**b**) Divergence of the same protocell: high *η_i_* (*α_i_* equal to 0.01, random *η_i_* with mean equal to 0.032).

Let us remark that, while in this work we present the results concerning protocells composed of 15 replicators and 15 precursors, we actually tested systems of very different sizes and in no case—out of thousands of matrices—did the equivalent “buffered” protocell model show synchronization (see Figure 7 for an example).

## 4. Conclusions and Indications for Further Work

The main outcome of this study is that of highlighting the importance of finite diffusion when the kinetic equations are nonlinear. The fact that all the different kinetic models which we have considered, which support the growth of the population of protocells, do actually synchronize, extends our previous conclusions on buffered models (where some divergences had been encountered) and makes a strong case in favor of the widespread appearance of spontaneous synchronization in early life.

In the past, we had already worked with some protocell models with finite diffusion, showing that they synchronized, but they were introduced either (i) to provide a “more realistic” system or (ii) to slow down the overall process [17]. In this paper, we provide a much more careful analysis of the way in which divergence occurs in buffered models, showing that it is due to an unrealistic “infinitely fast” inflow of permeable species, which provides a sound basis to resort to models with finite diffusion rates.

Moreover, in our recent studies it was possible to test that synchronization was always achieved in models of the kind we examined. We cannot claim that we have proven that synchronization happens for every conceivable set of kinetic equations, but this seems definitely to be the case whenever the equations are related to the law of mass action, i.e., when the reaction rates are proportional to frequencies of encounters of reactants, maybe raised to some non-integer exponent to account for complex reaction schemes.

Let us also remark that synchronization can spontaneously emerge when fission leads to two daughter protocells of equal size, perhaps perturbed by some random fluctuation, but it cannot emerge when the mechanism of cell division consistently leads to offsprings of different sizes, like in the case of budding. However, it has recently been proved that even in this case, sustainable population growth can occur, since the internal composition of the protocell (i.e., the ratios between the quantities of different types of replicators) is conserved in the case of buffered IRMs [31]. There is no reason to believe that this property does not hold when finite diffusion rates are taken into account, but this still has to be verified.

Last but not least, let us observe that the work presented here does not address the fundamental issue of understanding the evolution of control mechanisms which guarantee that cell reproduction takes place only after gene duplication, as happens in modern cells. This point is still open to further study, while our results support the idea that spontaneous forms of synchronization might have predated these more sophisticated mechanisms, allowing sustained population growth before they set in.

## Data Availability

The raw data supporting the conclusions of this article will be made available by the authors on request.

## Abbreviations

The following abbreviations are used in this manuscript:

DNA	Deoxyribonucleic Acid
SRM	Surface Reaction Model
IRM	Inner Reaction Model
